# Checkpoints That Regulate Balanced Biosynthesis of Lipopolysaccharide and Its Essentiality in *Escherichia coli*

**DOI:** 10.3390/ijms23010189

**Published:** 2021-12-24

**Authors:** Gracjana Klein, Alicja Wieczorek, Martyna Szuster, Satish Raina

**Affiliations:** Laboratory of Bacterial Genetics, Faculty of Chemistry, Gdansk University of Technology, 80-233 Gdansk, Poland; alic.wieczorek@gmail.com (A.W.); szuster.mart@gmail.com (M.S.)

**Keywords:** lipopolysaccharide, LpxC, MsbA, Kdo transferase, LPS assembly proteins LapB and LapC (YejM), acyltransferases

## Abstract

The outer membrane (OM) of Gram-negative bacteria, such as *Escherichia coli*, is essential for their viability. Lipopolysaccharide (LPS) constitutes the major component of OM, providing the permeability barrier, and a tight balance exists between LPS and phospholipids amounts as both of these essential components use a common metabolic precursor. Hence, checkpoints are in place, right from the regulation of the first committed step in LPS biosynthesis mediated by LpxC through its turnover by FtsH and HslUV proteases in coordination with LPS assembly factors LapB and LapC. After the synthesis of LPS on the inner leaflet of the inner membrane (IM), LPS is flipped by the IM-located essential ATP-dependent transporter to the periplasmic face of IM, where it is picked up by the LPS transport complex spanning all three components of the cell envelope for its delivery to OM. MsbA exerts its intrinsic hydrocarbon ruler function as another checkpoint to transport hexa-acylated LPS as compared to underacylated LPS. Additional checkpoints in LPS assembly are: LapB-assisted coupling of LPS synthesis and translocation; cardiolipin presence when LPS is underacylated; the recruitment of RfaH transcriptional factor ensuring the transcription of LPS core biosynthetic genes; and the regulated incorporation of non-stoichiometric modifications, controlled by the stress-responsive RpoE sigma factor, small RNAs and two-component systems.

## 1. Introduction

Gram-negative bacteria, such as *Escherichia coli*, are endowed with a cell envelope, which is comprised of an outer membrane (OM) and an inner membrane (IM) separated by the periplasm, containing a thin layer of peptidoglycan. The defining and most distinguishing feature of such didermic bacteria is the presence of conserved asymmetric OM, which is essential for their viability [1]. This asymmetric nature of OM is due to the presence of lipopolysaccharide (LPS) at the outer leaflet, with phospholipids facing its inner leaflet. Lipopolysaccharide constitutes the major component of OM. Under exponential growth conditions, approximately 2 × 10^6^ molecules of LPS cover nearly 75% of the cell surface [1,2,3]. The importance of LPS is manifested since it is the major virulence factor of pathogenic Gram-negative bacteria [2,3]. The interaction of negatively charged phosphate residues of LPS molecules with divalent cations, such as Ca^2+^ and Mg^2+^, and hydrophobic interactions between acyl chains leads to strong lateral interactions, which create a tight packing between LPS molecules. Thus, the overall chemical composition of LPS endows its properties that provide the permeability barrier function, thereby preventing the entry of various antibiotics, bulky hydrophobic detergents and toxic compounds, such as bile salts, into the bacterial cell [1]. LPS is a complex glycolipid comprised of a highly conserved hydrophobic membrane-anchored lipid A and a core oligosaccharide, which is linked to an oligosaccharide with variable lengths called the *O*-antigen in smooth-type bacteria [2]. LPS per se is highly heterogeneous in its chemical composition, and this heterogeneity can arise due to regulated differences in the acylation of lipid A part and the incorporation of non-stoichiometric modifications in lipid A and the LPS inner core [3,4,5,6]. The incorporation of these modifications is tightly regulated and requires inducing the expression of genes belonging to two-component systems (TCS), such as BasS/R, PhoB/R and PhoP/Q, and to RpoE regulon members, including its non-coding arm [7,8,9,10,11,12].

**Figure 1:**
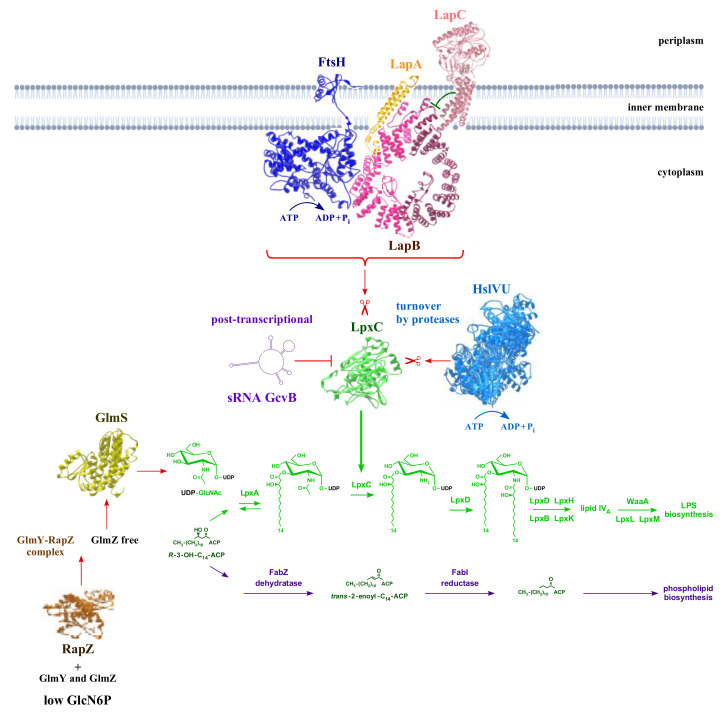
Regulation of the first committed step in LPS biosynthesis catalyzed by LpxC. On the top schematic drawing of the interaction between LapB and LapC in the inner membrane, and scissors depict LpxC proteolysis by FtsH and HslVU proteases. The LpxC synthesis is also subjected to negative post-transcriptional regulation by the GcvB sRNA. At the bottom, the recruitment of UDP-GlcNAc and (*R*)-3-hydroxymyristate precursors in LPS biosynthesis is shown. The same (*R*)-3-hydroxymyristate precursor is used by FabZ in phospholipid biosynthesis. UDP-GlcNAc synthesis requires GlmS, whose amounts are regulated by GlmY and GlmZ sRNAs, requiring RapZ as an adaptor protein.

The viability of all Gram-negative bacteria, including model bacteria such as *E*. *coli*, is contingent on the maintenance of a tight balance between phospholipids and LPS, which is held at a constant ratio of 1:0.15 [13]. Any imbalance in this composition is lethal for bacterial growth [14,15]. Thus, various cellular regulatory controls and checkpoints maintain this tight balance. In this review, we will discuss these checkpoints in *E*. *coli*. While other organisms have similar systems, specific details, such as the type and number of acyl groups attached to lipid A, differ among different species. The first regulatory control is exerted by balancing LPS vs. phospholipid amounts via the controlled turnover of LpxC, which catalyzes the first committed step in LPS biosynthesis (Figure 1) [13,14,15,16]. Additionally, the fact that LPS is synthesized continuously, and its translocation to the OM from the outer leaflet of IM requires ATP and uses an elegant transenvelope complex of seven essential Lpt (lipopolysaccharide transport) proteins, with its components present in all three compartments, are both of significance [17,18].

The biosynthesis of LPS, including its conserved lipid A part, occurs on the inner leaflet of IM. After the completion of LPS synthesis, it is then flipped by an essential ATP-dependent flippase known as MsbA to the outer leaflet of IM. To ensure that primarily LPS with hexa-acylated lipid A is translocated, MsbA provides an essential checkpoint as it selects species that are hexa-acylated by several orders of magnitude [19,20]. At this point, the viability of bacteria with either tetra- or penta-acylated lipid A requires the presence of cardiolipin (CL), which presumably aids in the transport of such LPS, constituting another checkpoint [21,22]. Once LPS is flipped by MsbA, it is delivered to the Lpt machinery for its final assembly in the OM. Dysfunction in any of the Lpt transenvelope components results in the accumulation of LPS at the periplasmic side of IM and is often modified by the M-antigen [17]. However, the Lpt system does not discriminate between the incompletely synthesized LPS and fully mature LPS. To ensure that the completely synthesized LPS is delivered to the Lpt system, the essential LapB protein provides an additional checkpoint by linking the LPS synthesis to its transport by acting as a scaffold for LPS biosynthetic proteins, ensuring that only the completely synthesized LPS is delivered to the Lpt complex [15]. Furthermore, to ensure that products of genes that are involved in the biosynthesis of the LPS core oligosaccharide are available, the transcription of the major *waaQ* operon, which encodes many of these proteins, requires the RfaH transcriptional factor [23]. RfaH reduces polymerase by pausing at a specific site in front of *waaQ* gene and thereby overcoming the premature transcriptional termination, which is critical for the expression of such operons as they lack the canonical ribosome-binding site [24]. The activity of RfaH is, however, inhibited by the interaction with a non-coding sRNA RirA, providing another checkpoint to prevent the accumulation of excessive LPS to maintain its balanced biosynthesis [23]. It is pertinent to point out that although LPS is essential for the majority of Gram-negative bacteria, there are few exceptions where some bacteria can survive without LPS (reviewed in [3]). In this review, we will discuss these processes in *E*. *coli*, with a particular emphasis on the regulation of the first committed step in LPS biosynthesis by LpxC as it is a topic of more recent studies with the discovery of the critical role played by the essential IM-anchored LPS assembly protein LapC, the identification of additional proteolytic control mechanisms exerted by HslUV protease at high temperatures and the regulation by the GcvB regulatory RNA (Figure 1).

## 2. Regulation of the Balance between LPS and Phospholipid Biosynthesis Flux of the Common Metabolic Precursor (*R*)-3-hydroxymyristate in Two Pathways

In *E*. *coli*, the first step in lipid A biosynthesis is catalyzed by the LpxA enzyme. LpxA mediates the transfer of a single acyl chain to UDP-GlcNAc, resulting in the synthesis of UDP-3-*O*-[(3R)-3-hydroxyacyl]-GlcNAc [2,3,25,26]. The identification of LpxA as a UDP-GlcNAc acyltransferase revealed that UDP-GlcNAc is situated at an early branch point, leading to the synthesis of either peptidoglycan or lipid A (Figure 1, Table 1) [5,27]. This LpxA-catalyzed reaction requires an (*R*)-3-hydroxy moiety of the fatty acyl-ACP as a substrate and, in *E*. *coli*, is selective for 14 carbon substrates [28]. This selectivity is a result of the LpxA active site performing a hydrocarbon ruler function that preferentially incorporates 14 carbon substrates [29,30]. The most remarkable aspect of LpxA’s acyltransferase activity is its unfavorable equilibrium constant [27]. Hence, the second irreversible reaction becomes the first committed step in lipid A biosynthesis, catalyzed by Zn^++^-dependent deacetylase LpxC, leading to the deacetylation of UDP-3-*O*-[(3R)-3-hydroxyacyl]-GlcNAc for the synthesis of UDP-3-*O*-[(3R)-3-hydroxyacyl]-GlcN, which in turn serves as a substrate for LpxD to yield UDP-2,3-diacylglucosamine [31,32,33,34,35]. This intermediate is then converted to the lipid IV_A_ precursor by the further action of LpxH, LpxB and LpxK (Figure 1) [2,26]. In this essential process, (*R*)-3-hydroxymyristoyl-ACP is situated at another important biosynthetic branch point since it can be elongated to palmitate in order to be incorporated into glycerophospholipids instead of recruited into lipid A biosynthesis [14]. As LPS and phospholipids share (*R*)-3-hydroxymyristate as a common metabolic precursor, a tight balance is maintained in the amounts of these two essential components of the cell envelope [14,36]. This is achieved by the regulation of the LpxC amount and FabZ dehydratase activity to maintain a flux of a common metabolic precursor in the utilization of LPS and phospholipid biosynthesis. Thus, the study of LpxC-mediated regulation has become an intense topic of investigation in recent years [37,38,39].

## 3. FtsH-Mediated Control of LpxC Turnover

Early studies hinted that the overproduction of LpxC cannot be maintained due to its toxicity, although the reasons for this phenomenon were not understood [40]. This can now be explained by the diversion of (*R*)-3-hydroxymyristoyl-ACP to lipid A biosynthesis at the expense of phospholipid synthesis, resulting in the depletion of glycerophospholipids and/or UDP-GlcNAc, which can be lethal. During seminal studies by Raetz laboratory that led to initial discoveries of the lipid A biosynthetic pathway, it was observed that the deacetylase activity provided by LpxC is elevated when lipid A biosynthesis is inhibited using either *lpxA* or *lpxD* mutants [27]. These findings suggested that LpxC is subjected to regulation in response to the lipid A content and/or synthesis rate. In a further extension of this work, it was shown that the inhibition of either the acyltransferase activity (LpxA-dependent) or the deacetylase results in a 5–10-fold increase in LpxC activity, which does not accompany any increase in the transcription of the *lpxC* gene, but there is an increase in the accumulation of LpxC [41]. Thus, these authors proposed two models invoking either a control at a translational level or proteolytic turnover of LpxC upon the accumulation of the disaccharide bisphosphate precursors of lipid A [41]. Indeed, our current level of understanding supports both models, although most of the attention has focused on proteolytic turnover (Figure 1) [21]. An important breakthrough was provided when FtsH was identified as the protease that mediates LpxC turnover and how this step of regulation establishes a critical checkpoint to have balanced biosynthesis of phospholipids and LPS [14]. These authors, while studying the essentiality of the *ftsH* gene, isolated a suppressor mutation called *sfhC* (for suppressor of *ftsH*), which suppresses the lethality of the *ftsH* mutation. FtsH is an essential AAA^+^ metalloprotease, which also regulates the turnover of many substrates that include the RpoH heat shock sigma factor [14]. Further characterization of the s*fhC21* mutation revealed it has a mutation in the *fabZ* gene [14]. The *fabZ* gene encodes for the (*R*)-3-hydroxyacyl-ACP dehydratase, and the encoded enzyme also uses (*R*)-3-hydroxymyristoyl-ACP [13,42], which, as mentioned above, is situated at an important branch point in the synthesis of lipid A and phospholipids. Besides establishing that LpxC is a substrate of FtsH, these studies also showed that *ftsH* mutations cause a 5–10-fold increase in the accumulation of LpxC deacetylase, resulting in the increased synthesis of LPS, which leads to the lethality and, thus, the essentiality of FtsH [14]. The rationale of suppressing *ftsH* mutations by *sfhC21* is explained by enhanced FabZ dehydratase activity, thereby compensating for high levels of deacetylase when FtsH is absent.

So far as the mechanism of LpxC recognition is concerned, it has been shown that six non-polar residues in its C-terminal tail of about 20 amino acids are critical for degradation by FtsH [43]. This proteolysis of LpxC is controlled by the cellular growth rate and levels of alarmone ppGpp [44]. Thus, LpxC is rapidly degraded under slow-growth conditions and is relatively more stable under fast-growth conditions, and this phenomenon is reversed when ppGpp is absent [44]. However, how FtsH activity is regulated in response to the accumulation of LPS remained unknown until the discovery of LPS assembly proteins LapA/LapB and LapC [15,38].

## 4. LapB Functions to Regulate FtsH-Mediated Proteolysis of LpxC and Determine the Major Checkpoint of Regulating LPS Biosynthesis in Concert with FtsH

To identify any additional factors that regulate the LPS content, mutations that cause a constitutive induction of the RpoE sigma factor, which acts as the main cell envelope’s stress regulator and sensor, were isolated [15,45]. Such a phenotype is reminiscent of mutations in genes that affect either the LPS synthesis or its translocation since one of the potent signals of induction of an RpoE-dependent cellular stress response is related to sensing LPS defects [23]. Through further characterization of the transcriptional regulation of genes, their non-polar disruptions and analysis of LPS content of isolated mutants identified an operon comprised of *lapA* and *lapB* genes. Among these two genes, the *lapB* gene was found to be essential for bacterial growth, and the deletion of this gene could only be tolerated on minimal medium at 30 °C in certain backgrounds, but not in the majority of wild-type strains unless supplemented by the plasmid-born wild-type copy of the gene or in the presence of extragenic suppressors [15]. Significantly, the analysis of LPS and suppressors of Δ*lapB* or Δ(*lapA lapB*) revealed that such strains synthesize an excess of LPS and that suppressor mutations in either the *lpxC* gene or in those genes that reduce the LPS content can overcome the lethality of bacteria in the absence of LapB [15]. This phenotype mimics the phenotype of *ftsH* mutants, and, not surprisingly, the introduction of the *sfhC21* hyperactive allele of *fabZ* also bypasses the lethality of Δ*lapB* just like that of Δ*ftsH* [14,15]. More evidence of LapB’s participation in regulating LpxC amounts, and thereby LPS biosynthesis, came from the biochemical evidence showing a physical interaction between LapB and FtsH and the co-purification of LPS with either LapA or LapB [15]. As observed with *ftsH* mutants, a Δ*lapB* mutation can be tolerated when lipid A biosynthesis is reduced due to a mutation in either *lpxA* or *lpxC* or *lpxD* genes or in the early steps of LPS inner core biosynthesis [15]. This was based on the identification of suppressor mutations in either the *lpxC* gene (causing a reduction in LpxC amounts), a mutation in either heptose biosynthesis (disruption of *gmhA*) or a single amino acid alteration WaaC T187K (located within the sugar-nucleotide binding site resulting in a loss of function of WaaC) [15]. The *gmhA* gene encodes sedoheptulase 7-phoshaphate isomerase, which catalyzes the first committed step in heptose biosynthesis, and the *waaC* gene encodes heptosyl transferase I, which transfers the first heptose to the Kdo_2_ moiety of the LPS’s inner core [46,47]. Similarly, a decrease in the *waaQ* operon expression, which carries several co-transcribed genes whose products are involved in LPS core biosynthesis, can reduce LPS biosynthesis in a negative feedback manner to counterbalance the effect of increased LpxC in Δ*lapB* mutants [15]. It is quite plausible that in Δ*lapB*, an excess of LPS accumulates in the inner membrane and contributes to toxicity. Support for such a notion comes from the presence of aberrant LPS species in Δ*lapB* mutants (underacylated or altered lipid A and core composition) [15]. Thus, mutations that reduce the LPS synthesis due to the hyperdegradation of LpxC led to the identification of another partner of LapB, designated LapC, that acts in an antagonistic manner, bypassing the LapB essentiality (see below) [38]. Similarly, downregulating the synthesis of the major murein protein Lpp, the most abundant protein in *E. coli*, and the suppression of the hyperelevated envelope stress response due to the overexpression of a novel small non-coding RNA SlrA (MicL) also bypass the essentiality of LapB [15]. The SlrA-mediated suppression of Δ(*lapA lapB*) is explained by its translational repression of *lpp* [15,48]. This is consistent with the isolation of the loss-of-function mutation in the *lpp* gene, which overcomes the lethality of *lapB* deletion. A loss of Lpp function results in the altered permeability of OM and hypervesiculation, which can relieve the toxic accumulation of LPS in the IM as vesicles are known to contain LPS, and their shedding could decrease the LPS content [15,49,50]. Independent studies aiming to study the temperature-sensitive (Ts) phenotype of a Δ*lapB* gene (*yciM*) derivative from the Keio collection revealed that such a strain was viable due to a suppressor mutation in the *lpxC* gene and that depletion of LapB results in the increased accumulation of LpxC and LPS [51]. Thus, in the absence of either FtsH or LapB, cellular toxicity stems from the excess of LPS synthesis at the expense of phospholipids due to the increased accumulation of LpxC, which results in the depletion and diversion of common metabolic precursor (*R*)-3-hydroxymyristoyl-ACP as well as the accumulation of toxic levels of LPS in the IM. This also explains their essentiality, which can be overcome by either the increased activity or higher dosage of the FabZ enzyme.

## 5. LapB Coordinates the LPS Synthesis and Translocation

Analysis of LPS composition and pull-down experiments highlight that LapB could act as a scaffold protein in the inner membrane to recruit various LPS biosynthetic enzymes to ensure that only the completely synthesized LPS is delivered to LPS transport proteins. Support for this idea comes from an accumulation of immature species of LPS in strains lacking LapB, which has not been reported as a result of the loss of FtsH. In addition to the accumulation of several early intermediates of LPS biosynthesis, lipid A of Δ*lapB* strains was also found to contain penta-acylated species and species with two lauroyl chains with an additional lauroyl chain located in the same place where the myristoyl chain is usually added [15]. Furthermore, LapB was found to co-purify with WaaC heptosyltransferase, Lpt proteins and various LPS glycosyltransferases in addition to FtsH and LPS [15]. Additional evidence for this comes from the observed aggregation of LpxM, WaaC and WaaO in the absence of LapB, which can result in LpxM becoming limiting. Thus, the aggregation of LpxM can explain the accumulation of penta-acylated lipid A species as well as the incorporation of an additional lauroyl chain.

**Figure 2:**
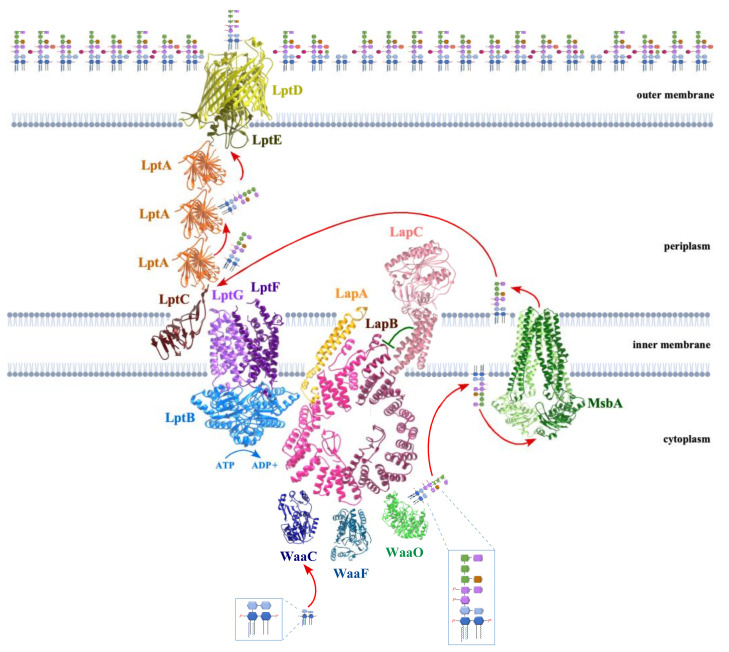
LapB–FtsH-mediated proteolytic turnover of LpxC is regulated by LapC, and LapB couples the LPS synthesis with its translocation. In this process, LapC acts as an antagonist of LapB, preventing unwanted proteolysis of LpxC. Model of LPS synthesis with LapB acting as a scaffold to assemble LPS and couple the LPS synthesis to its translocation by the Lpt system. MsbA flips LPS after the completion of its assembly on the inner side of IM to the outer leaflet of IM for its delivery to Lpt proteins. Based on interactions of LapB with WaaC and other LPS biosynthetic enzymes, some of them are depicted in this scheme of LPS assembly.

The co-purification of LapA and LapB with LPS, WaaC, FtsH and Lpt transport proteins provides strong evidence that LapB, probably along with LapA, acts as a scaffold in the IM that could allow recruiting various enzymes for the completion of LPS synthesis and deliver mature LPS to the Lpt system (Figure 2). LapB has nine TPR (Tetratricopeptide Repeat) elements, and many conserved amino acid residues have been shown to be essential for its function regulating LpxC proteolysis [15,52]. TPR repeats could mediate the protein–protein interaction and help recruit different LPS biosynthetic proteins at the site of LPS synthesis. In this process, LapB by dint of its proposed scaffold function can simultaneously present LpxC to FtsH for proteolysis. Suppressor analysis also revealed that overexpression of chaperone-encoding genes (*dnaK*, *dnaJ*) could partially mitigate LapB essentiality [15]. Not surprisingly, DnaK and DnaJ were also found to co-purify with LapA and LapB proteins, and Δ(*dnaKJ lapA lapB*) mutants exhibited even more severe LPS defects. Thus, it was proposed that DnaK and DnaJ chaperones could maintain cytoplasmic and IM-associated LPS biosynthetic enzymes in a folding competent manner and deliver functional enzymes to LapB for completion of LPS biosynthesis. Further evidence that LapA and LapB couple the LPS synthesis and LPS transport comes from the synthetic lethality of Δ(*lapA/B*) combination with *lptD* dysfunctional alleles (Figure 2) [15]. As LptD is a substrate of SurA periplasmic protein, deletion of the *surA* gene is also not tolerated in Δ*lapB*.

It was further found that the lethality of LapB absence can be overcome by overproduction of MurA, which uses UDP-GlcNAc for the peptidoglycan synthesis [15]. This is best explained by excess MurA’s ability to shift the utilization of the UDP-GlcNAc precursor towards peptidoglycan balance and reduce the LPS synthesis by limiting its availability to LpxA, which, in turn, can reduce the excess LPS synthesis in Δ(*lapA*/*B*) derivatives. This is consistent with the fact that the biosynthesis of LPS and peptidoglycan use UDP-GlcNAc as a common metabolic substrate, its utilization constitutes the first branch point. Hence, the regulation of this precursor’s synthesis plays an important role and requires a balanced synthesis of GlmS according to the availability of UDP-GlcNAc (Figure 1). This step is regulated by GlmY/Z sRNAs and the RapZ adaptor protein (for details see [5,53].

## 6. Regulation of LapB/FtsH Proteolytic Control of LpxC by LapC

It has remained a moot point how FtsH and LapB sense LPS amounts and trigger proteolysis of LpxC since an unchecked decrease in the amount of LpxC can reduce the LPS synthesis and lead to toxicity. Several studies in the last year revealed that essential protein LapC (previously YejM) acts as an antagonist of LapB and could serve as an essential checkpoint in maintaining a critical concentration of LpxC as per cellular demand and sense the accumulation of LPS (Figure 1). The discovery of LapC began by the isolation of a Ts strain LH530 with an unknown mutation conferring a reduction in LPS amounts with a concomitant sensitivity to fusidic acid antibiotic [54]. Sensitivity toward hydrophobic drugs and the reduced LPS content implied that the mutation could alter the permeability barrier function of OM. Though the mutation in LH530 was not identified, it could be phenotypically suppressed by the overexpression of the *acpT* gene, which encodes a holo-acyl carrier protein synthase. Further characterization of the LH530 strain revealed that it carried a mutation in the essential *yejM* gene, resulting in the truncation of its entire periplasmic domain [55]. YejM was found to contain the essential IM domain with five transmembrane helices and a dispensable periplasmic domain. The truncation of the periplasmic domain was found to confer antibiotic sensitivity and breach the OM barrier function, although its role in the regulation of LPS was not known [54,55]. Subsequent studies on *Salmonella enterica* serovar Typhimurium (*S.* Typhimurium) suggested LapC’s function (called PbgA) in enhancing the OM integrity and its requirement for survival in macrophages and mice based on the isolation of mutations that lead to the truncation of its non-essential periplasmic domain [56]. The authors suggested that LapC is required for changes in the OM barrier’s function, which is controlled by PhoP/Q TCS and hence designated PbgA (PhoPQ-Barrier Gene A) [56]. Furthermore, related to PhoP/Q activation, it was found that such inducing conditions lead to the increased accumulation of cardiolipins [57]. As the cardiolipin content did not increase in *lapC* mutants, it was hypothesized that LapC could mediate cardiolipin transport to the OM. However, such a function of LapC in the cardiolipin transport awaits experimental support since LapC does not exhibit any similarity to transport proteins.

The first hint for LapC function in regulating LPS biosynthesis came from studies with *lapC* (*pbgA)* mutants in *Salmonella* while examining survival in the mice model system when challenged with either the wild-type or *lapC* mutants. While the wild type readily kills mice, *lapC* mutants were attenuated in infection, and *lapC* mutants that survived had suppressor mutations mapping to either *ftsH* or *lapB* or *lpxC* [58]. As suppressor mutations mapped to regulators of the first committed step of LPS biosynthesis, it allowed authors to suggest that LapC could somehow regulate LPS biosynthesis, which was supported by changes in the LPS content [58]. The main breakthroughs in understanding the LapC function in regulating LPS biosynthesis came from five independent studies, although some of these studies were not aimed at addressing the regulation of LpxC degradation by FtsH–LapB [38,59,60,61,62]. Nonetheless, all these studies suggest a role for LapC in regulating the turnover of LpxC.

Exploiting phenotypic defects of a strain lacking the periplasmic domain of LapC, extragenic suppressors were isolated, and their sequencing identified three mutations mapping to the *lapB* gene and two mutations in the *lpxC* gene [61]. Suppressors in either the *lapB* gene or the *lpxC* gene were found to increase the LPS amount, suggesting LapC could regulate LapB–FtsH mediated LpxC degradation [61]. Moreover, mutations that reduce the LPS synthesis were found to be lethal in such *lapC* mutant bacteria and in contrast, inactivation of LapB rendered LapC dispensable. This allowed the authors to conclude that LapC functions upstream of LapB to control LpxC degradation [61]. In a similar study, suppressors that overcome the sensitivity of *lapC* mutants to bulky antibiotics, such as vancomycin and erythromycin, were isolated [60]. Sequencing analysis of such six antibiotic-resistant isolates revealed the presence of a mutation either in the *lapB* gene or in the *ftsH* gene, and such suppressor mutations restored LpxC levels [60]. These authors hypothesized that LapC could interact with FtsH to regulate LpxC degradation, although this model awaits verification. Another study aimed to address the essentiality of the *lapC* gene performed depletion experiments [62]. Suppressors that tolerated the depletion of LapC were mapped to the *lpxC* and *lapB* genes, and overproduction of LpxC was found to allow the survival of Δ*lapC* bacteria. Significantly, LpxC V37G and LapB H181R were found to allow the depletion of LapC. Indeed, this set of suppressors was independently identified as a part of a large pool of suppressors of *lapC* mutants lacking the C-terminal domain (see below for details) [38]. Consistent with observations from other groups, this study also showed that the absence of functional LapC results in decreased amounts of LpxC due to its elevated degradation. Using image analysis designed to map localization and interaction of membrane proteins, authors found an interaction between LapB and LapC but not of LapC with FtsH [62].

## 7. Mutations That Simultaneously Confer Sensitivity to CHIR090 and Induce Transcription from LPS Defects-Responsive *rpoE*P3 Promoter Identify the *lapC* Gene

In an exhaustive study that aimed to investigate the essentiality of LapB, identify additional partners of LapB–FtsH complex that regulate LpxC proteolysis and determine whether LapB–FtsH independent proteolysis exists, multipronged approaches were taken that revealed an antagonistic action of LapC on LapB [38]. In one approach, extragenic suppressors that bypass the lethality of a *lapB* deletion, resulting in the restoration of a normal level of LPS synthesis in a Δ*lapB* strain with a complete LPS core, were analyzed. One such suppressor mutation was shown to have a frame-shift mutation after amino acid residue 377 in the *lapC* gene, resulting in a truncation of the remaining periplasmic domain. Analysis of LPS of (Δ*lapB lapC377fs*) revealed increased amounts of LPS as compared to dramatically reduced LPS levels in *lapC377fs* and the suppression of the accumulation of LPS precursors as compared to their elevated levels in Δ*lapB*. Thus, phenotypically, while a *lapB* deletion results in elevated levels of LPS, but the truncation of the periplasmic domain of *lapC* results in its reduction, which prompted authors to conclude that LapB and LapC act in an antagonistic manner to control LPS biosynthesis [38]. In a direct approach to identifying regulators of LPS synthesis at the level of control of LpxC regulation, Ts mutations that simultaneously resulted in LPS defects (monitored by *rpoE*P3 promoter activation) and hypersensitivity to sub-lethal concentrations of LpxC inhibitor CHIR090 were isolated. CHIR090 is a known potent inhibitor of LpxC [63,64]. The rationale for this screen was based on the assumption that such a regulator of LPS biosynthesis should be an essential gene, and a dysfunctional derivative of such a candidate gene should result in LPS-related permeability defects, induction of *rpoE*P3 promoter and sensitivity to CHIR090 if LpxC is destabilized. This comprehensive study resulted in the isolation of mutations mapping to the *lapC* gene: one causing a truncation of the entire periplasmic domain *lapC190* and the repeated isolation of the *lapC377fs* mutation and one with a single amino exchange (*lapC* F349S) in the periplasmic domain of LapC. Among these, *lapC190* and *lapC377fs* conferred a tighter Ts phenotype as well as hypersensitivity to CHIR090 and were found to have lower amounts of LpxC and LPS. These phenotypes were used to isolate extragenic suppressors, which mapped to structural genes of *lpxC*, *lapA*, *lapB*, *ftsH* and to the promoter region of *lapA*/*B* operon. This suppressor screen was quite exhaustive based on the mapping of 26 suppressors; hence, many mutations were isolated several times, for example, the exchange of V37L/G, R230C in LpxC and the same spot-of-insertion element disrupting the *lapA* coding region. All these suppressors resulted in the stabilization of LpxC and restoration of LPS levels in *lapC* mutants. As transcription and translation of *lapA* and *lapB* genes are coupled, most of the analyzed *lapC* mutant strains with suppressor mutations mapping to the *lapA*/*B* operon had the reduced accumulation of LapB, explaining the enhanced stability of LpxC and hence suppression of *lapC* mutants that have less LPS and less LpxC. Several independently isolated suppressor mutations in the *lapB* gene had a single amino acid exchange in the highly conserved residues in TPR elements of LapB. Such mutations could prevent the interaction with proteins such as FtsH or the interaction with its own rubredoxin domain, therefore inhibiting LapB activity that rescues *lapC* mutants. Consistent with a working model of LapC acting in an antagonistic manner to prevent excessive degradation of LpxC by the LapB–FtsH complex, a suppressor mutation mapping to the *ftsH* gene was located in the conserved domain of FtsH, which is known to be required for its catalytic activity and may be needed for the stability of FtsH as well. Thus, a *lapC190 ftsH* A296V variant was found to have a reduced amount of FtsH as compared to the parental wild-type or a *lapC190* variant and, together with presumed reduced ATPase activity, could prevent LpxC proteolysis. As *lapC377fs* was originally identified as a suppressor mutation of Δ*lapB* that relieved its lethality, and most of the suppressor mutations of either *lapC190* or *lapC377fs* mapping to the *lapA*/*B* operon had reduced LapB amounts, and as an extension of these observations, LapB was shown to be dispensable in Δ*lapC,* and the overexpression of *lpxC* allowed the deletion of the *lapC* gene. Thus, the results from this study and the other studies mentioned above point to LapC’s role in controlling LpxC degradation by preventing its unwarranted degradation by the LapB–FtsH complex under conditions when higher amounts of LPS synthesis are required (for example, under fast-growing conditions and at higher temperatures). In support of such a model, it was further shown in pull-down experiments that LapB and LapC co-purify [38]. This is consistent with image analysis, showing their co-localization and LapC-Flag’s interaction with LapB in parallel studies [59,62].

## 8. How Demand for the LPS Synthesis Is Sensed by LapC and Impacts Its Interaction with LapB

**Figure 3:**
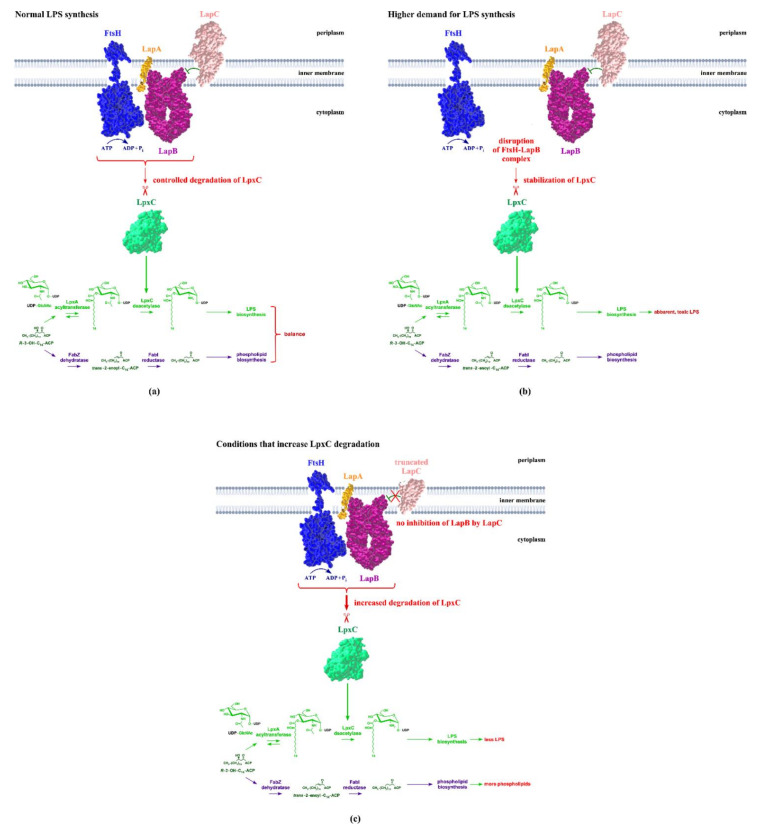
Model of LapC-mediated control of LpxC degradation as a function of the demand for LPS synthesis. (**a**) Under normal-growth conditions, LpxC and FabZ activity is coupled, maintaining a balanced synthesis of LPS vs. phospholipids. (**b**) When the demand for LPS synthesis is high, such as under fast-growing conditions, LapC inhibits and traps LapB preventing the LapB–FtsH complex formation and hence preventing excessive degradation of LpxC. (**c**) When the LapC periplasmic domain is absent, the LapB–FtsH complex is hyperactive, leading to increased degradation of LpxC. Similarly, when the demand for LPS is less, such as under slow-growth conditions or in the stationary phase, LPS accumulates in the periplasm, leading to increased destabilization of LpxC, with LapC not inhibiting LapB effectively.

While studying the growth rate-related LpxC degradation and ppGpp’s involvement, it became apparent that the slow-growth rate leads to a faster turnover of LpxC [44]. However, in strains that do not synthesize ppGpp, this growth rate-dependent LpxC proteolysis is reversed, leading to LpxC destabilization under fast-growth conditions and vice versa under slow-growth conditions [44]. In a search for identifying interacting partners of LpxC and FtsH, many proteins mostly involved in either LPS, fatty acid or phospholipid metabolisms were found to modulate the rate of LpxC proteolysis [65]. How LapC could be involved in regulating LpxC degradation and LPS synthesis still remained a mystery. Quantification of *lapC* transcripts under slow-growth conditions (30 °C) vs. fast-growth conditions (shift to higher temperature) provide a possible answer for LpxC turnover by LapC (Figure 3a,b) [38]. As the abundance of *lapC* mRNA increases by more than 3-fold at higher temperature (fast-growing conditions when LPS demand will be more), it has been proposed that the increased presence of LapC could dampen LpxC turnover due to trapping of LapB by LapC. This can lead to a reduction in LapB–FtsH-mediated proteolysis [38]. This model gains experimental support from the following: (a) depletion of LapC or its dysfunction causes destabilization of LpxC, while the increased expression of LapC stabilizes LpxC; (b) LapC directly binds to LapB based on co-purification and localization but not with FtsH [38,59,62,66]; (c) Overexpression of *lpxC* bypasses the *lapC* essentiality, whereas *lapB* overexpression does not [38,59]. Further evidence establishes that LapC senses LPS as LapC co-purifies with LPS, and an interacting region in the interfacial domain (IFD) between the IM and the periplasmic domain has been shown based on structural analysis and mutagenesis [59]; (d) Significantly, the LapC T213D mutation in the IFD domain that is expected to disrupt LPS binding, increase LpxC levels and disrupt the OM homeostasis. Furthermore, depletion of LPS translocation across the IM by inhibiting MsbA resulted in increased levels of LpxC. Consistent with a role of sensing LPS accumulation in the periplasm, the inhibition of LptD, which can lead to LPS accumulation in the periplasm, decreases LpxC, suggesting the FtsH–LapB-mediated LpxC proteolysis is triggered [59]. Consistent with such a role, LapB’s IM domain was shown to interact with LapC via its TM region using a bacterial two-hybrid system [66]. Thus, these studies posit LapC as the sensor for the accumulation of LPS on the periplasmic side, which can arise when LPS synthesis and translocation coupling is disturbed or when the demand for LPS is less, for example, under slow-growth conditions, which can trigger LpxC proteolysis by the activation of the LapB–FtsH complex (Figure 3c). However, when the demand for LPS is higher, production of LapC is induced (high-temperature or fast-growing conditions), LapB and LapC could bind tightly in an LPS-free manner, which could prevent FtsH-mediated proteolysis of LpxC (Figure 3b). Despite these advances, there are certain remaining issues on the signal transmission as Lap-B–LapC interaction was found to persist even in the absence of IFD or truncation of periplasmic domains of LapC.

## 9. Structural Studies of LapC and LapB Reveal LapC Recognition of Lipid A and Essential Domains of LapB

So far, five independent studies have resolved the structure of LapC (either of full length or of the periplasmic domain alone), and LapB has also been described. The first study solved the structure of the periplasmic domains of *S.* Typhimurium and *E*. *coli lapC* residues 245–586. This globular domain showed a structure that resembled the family of the arylsulfatase proteins and lipoteichoic acid synthase LtaS of *Staphylococcus aureus* [67]. However, the LapC globular domain lacks the required residues for such enzymatic activity and was not found to bind metal ions [67]. These authors implicated a cardiolipin binding based on a previously purported role [56] and the co-sedimentation of the LapC globular domain with cardiolipin in in vitro assays [67]. This structural work was followed by the crystallization of full-length LapC from *S.* Typhimurium with premixed cardiolipin, and the authors assigned two cardiolipin-binding sites [68]. One of the cardiolipin-binding sites was located at the junction of transmembrane (TM) regions 1 and 2 of LapC, and the second binding site was positioned at the membrane interface. The second cardiolipin-binding site in LapC contacts showed conserved R215 and R216 amino acid residues [68]. The authors proposed that a deep cleft on the surface of the protein extending from the TM domain into the periplasmic domain could allow lipid substrates to enter or exit in support of the model for LapC’s involvement in cardiolipin transport [68]. However, genetic studies with a strain lacking all three cardiolipin encoding genes do not show a similarity in phenotypes with truncations in the LapC periplasmic domain [69]. More convincing and direct evidence for a role in LPS binding for LapC rather than cardiolipin transport came from the additional structural and mutational analysis [59]. This structural analysis established that LapC contains N-terminal transmembrane helices consisting of five TMDs, upon which the C-terminal periplasmic part sits [59]. The interfacial domain was found to be compacted, connecting TMD and the periplasmic domain, with the periplasmic domain only protruding 60 Å, ruling out shuttling cardiolipins to the OM [59]. Despite structural similarities with EptA and LtaS, a lack of catalytic and metal-binding residues in the periplasmic LapC region thus suggested it has a pseudo-hydrolase domain. The co-purification of LPS with LapC and the identification of lipid A-binding residues interacting with the helix containing amino acids from 210 to 217 in the IFD provide a promising model of LapC functioning. Thus, unlike other lipid A-binding proteins, LapC was found to uniquely recognize a single phosphorylated GlcNAc unit of lipid A. This interaction was supported by the introduction of point mutations in this lipid A-binding motif, revealing that mutations that can disrupt lipid A’s binding of LapC cause OM defects, such as sensitivity to antibiotics such as rifampicin. These results were supported by showing that a synthetic peptide derived from the lipid A-binding motif from the IFD sequence can bind LPS, and further, such peptide derivatives could effectively kill Gram-negative bacteria [59]. Taken together, these structural data [59] combined with the characterization of suppressors that overcome the lethal phenotype of Δ*lapB* strains mapping to the periplasmic domain of LapC, coupled with co-purification of LapB–LapC, provide support for LapC’s function in the recognition of LPS and an antagonistic action of LapC on LapB [38,59]. Finally, the last crystal structure reported for the LapC periplasmic domain of *S.* Typhimurium was unexpectedly identified as a metalloenzyme with magnesium-dependent phosphatase activity [70]. This phosphatase activity required the intact active site, five TMDs and the linker region. However, how this putative phosphatase activity of LapC is relevant to the control of LapC interaction with LapB in the control of LpxC levels is unclear. Interestingly, a single amino acid mutation *lapC*F349S located in the predicted phosphatase active site and Mg^2^+-binding pocket does not confer sensitivity to CHIR090 and exhibits a leaky Ts phenotype with much-reduced impact on the *rpoE*P3 promoter activity as compared to *lapC190* or *lapC377fs* mutants [38]. Thus, further investigation is required to connect any role of this putative phosphatase activity with LPS sensing by LapC.

Concerning LapB, modelling and crystal structure data reveal that it is anchored to the IM via its N-terminal domain and the rest of the protein is located in the cytoplasm [15]. The N-terminal cytoplasmic part contains nine TPR motifs, and the C-terminal part contains a rubredoxin domain and all three parts: the membrane anchor, TPR repeats and the rubredoxin domain are essential for its function [15,52,66]. In the initial identification of the *lapB* gene, several single amino acid exchanges in either TPR or rubredoxin domains resulted in its inactivation [15]. The structural analysis supported their isolation and also revealed that the rubredoxin domain is intimately bound to TPR motifs, covering more than 31% of its surface area [52]. Of significance, mutations that could prevent the docking of the rubredoxin domain on the TPR superhelix were found to result in loss of function of LapB. One such alteration is of H181, which was examined during a structure-function analysis of LapB and later on during the selection of suppressors of *lapC* mutants lacking its periplasmic domain [38,52]. TPR repeats are ideally used for protein–protein interactions [71] and could be used as a scaffold to recruit LPS-specific enzymes, including LpxC, for the regulated LPS synthesis [15]. The importance of all three domains for LapB was further shown by establishing that LapC and LapB TM anchors are required for their interaction [59,66]. However, despite the structural and genetic interactions between LapB and LapC, it remains unclear what triggers the disruption of this interaction, which directs the LapB–FtsH complex-driven proteolysis of LpxC.

## 10. LpxC Alternative Proteolysis-Regulated Turnover by Heat Shock Induced HslVU Protease Complex

After the discovery of the involvement of FtsH in the turnover of LpxC in *E. coli*, it was presumed that it is the only protease that is involved in this process [14]. Subsequently, it was shown that FtsH requires LapB function to mediate LpxC turnover and LapC can antagonize LapB to maintain a balance between the LPS synthesis and turnover process (see sections above). In a study that aimed to address the essentiality of LapB, multicopy suppressors that bypass the lethality of Δ*lapB* bacteria led to the discovery that LpxC can be a substrate for HslV protease, and this process is accelerated when its partner HslU is also present [38]. One of the suppressing plasmids was found to carry the gene encoding the HslV subunit of the HslUV protease complex. HslV is the catalytic subunit of this complex, and this proteolytic complex constitutes a prokaryotic counterpart of the eukaryotic proteasome [38,72]. To understand the biochemical basis of HslVU overexpression, it was shown that the overexpression of either *hslV* alone or of *hslVU* substantially reduces the accumulation of LpxC in the Δ*lapB* derivative, thus revealing that HslVU can mediate the turnover of LpxC in the absence of the LapB–FtsH complex [38]. These results were further supported by pulse-chase experiments, following the induction of HslVU and shutting off host protein synthesis, which showed a rapid turnover of LpxC at high temperatures (Figure 1). It needs to be emphasized that the expression of *hslV* and *hslU* genes, also called *clpQ* and *clpY*, respectively, is regulated by the RpoH heat shock sigma factor, and they are among the major heat-shock-inducible proteins [72,73,74,75]. Consistent with their transcription being induced at high temperatures, it has also been shown that HslVU’s proteolytic activity towards its substrates is enhanced at high temperatures [76]. This can also explain the restoration of the growth of Δ*lapB* or Δ*lapAB* strains at elevated temperatures. Consistent with the independent proteolysis of LpxC by FtsH–LapB, it was found that Δ*lapB* or Δ*lapAB* mutations can be introduced readily when *hslVU* genes are overexpressed. In line with a role for the HslVU-mediated regulation of LpxC turnover, Δ*hslV* and Δ*hslVU* were found to be sensitive to the LpxC inhibitor CHIR090 [38]. Thus, LapB becomes dispensable when either LapC is dysfunctional or when an alternative pathway of LpxC proteolysis mediated by HslVU is induced. A role for this alternative proteolytic control of LpxC independent of LapB–FtsH could be a backup mechanism to prevent the excessive synthesis of LPS under fast-growing conditions, such as at high temperatures, when FtsH-mediated turnover is reduced (Figure 1) [38,44]. It is not surprising to have additional proteolytic control of LpxC by HslVU in addition to FtsH, as some substrates, such as RpoH, are also subjected to a dual mode of degradation by FtsH and HslVU [38,75]. Similarly, other HslVU substrates, such as RcsA and SulA, are also subjected to the dual mode of regulation while under proteolytic control by Lon [74,75]. Overall, the identification of HslVU-mediated proteolytic control of LpxC independent of LapB–FtsH reveals a network of pathways that controls the first committed step in LPS biosynthesis.

## 11. Negative Regulation of LpxC by the GcvB sRNA-Potential Transcriptional Control

Small non-coding RNAs (sRNAs) are known to regulate the expression of several genes in most organisms [5,77,78]. They act by either base-pairing with mRNAs or through the sequestration of regulatory proteins [77,78]. sRNAs can regulate gene expression either negatively or positively. Thus, base-pairing sRNAs that negatively regulate the gene expression can occlude the ribosomal binding site to cause translational repression, or some sRNAs can also base-pair in the coding region, enhancing mRNA degradation [79,80,81]. Quite like many metabolic pathways, biosynthesis and modifications of LPS are also subjected to regulatory controls by some specific sRNAs (for recent reviews concerning LPS and sRNA-mediated control, see [3,5,82]). Here, we mainly address the regulation of *lpxC* expression by sRNAs. Using a library of plasmids carrying different sRNAs, the overexpression of the *gcvB* was found to allow deletion of the *lapB* gene, quite like that previously observed when SlrA (MicL) is overexpressed. GcvB is one of the best-studied sRNAs, and a plethora of interacting mRNAs have been identified [83,84]. GcvB regulates the expression of more than 50 genes in *E*. *coli*, and many of them encode amino acid transporters, metabolic enzymes and a few transcriptional regulators, including PhoP [83,85,86,87]. Interestingly, levels of GcvB are higher when bacteria grow in the exponential phase and decrease during the stationary phase [83,88]. Consistent with GcvB overexpression preventing the lethality in the absence of LapB, it has been suggested that this sRNA could modulate LpxC levels either directly or indirectly (Figure 1) [21]. Indeed, levels of LpxC are reduced when GcvB is overexpressed in the wild type, and a Δ*gcvB* mutation confers hypersensitivity to the LpxC inhibitor CHIR090. However, it is unknown if GcvB acts directly by the translational inhibition of *lpxC* mRNA or by a regulatory control of some other gene(s), whose product(s) are involved in the turnover of LpxC. However, it is clear that, while SlrA negatively regulates the Lpp synthesis and thereby alters permeability barrier properties, the mode of action of GcvB participation needs more studies.

## 12. The Cross-Talk between Various Intricate Pathways Linking Lipid and LPS Synthesis That Controls LpxC Levels

As mentioned in the above sections, mutations in *lpxA*, *lpxC* and *lpxD* genes that decrease the LPS synthesis result in the stabilization of LpxC, suggesting the presence (accumulation) of lipid A disaccharide on the cytoplasmic side after the LpxD action is sensed to determine the rate of LpxC degradation [32,41]. Consistent with such a model, reducing the accumulation of lipid A disaccharide by *lpxK* overexpression leads to the stabilization of LpxC [89]. However, many other signals also alter LpxC stability. Some of them are linked to the fatty acid metabolism involving fatty acid biosynthesis (*fab* genes) or its degradation (*fad* genes), suggesting that acyl-ACP pools have been sensed, and metabolic products, such as acyl-CoA, are produced by the conversion of fatty acids and activity of FabZ, FabA and FabI [39]. Thus, acyl-CoA has been shown to inhibit LpxC proteolysis [90]. Consistent with the link with fatty acid biosynthesis, overproduction of the FadR master regulator destabilizes LpxC [65]. It is known that the binding of FadR to its cognate promoters activates the transcription of *fad* genes and represses the FAB pathway [91]. Adding to this complexity, the ratio of saturated to unsaturated fatty acids is subjected to the interplay among isomerization, condensation and enoyl reduction reactions catalyzed by FabA, FabB and FabI, respectively [92,93]. Computational modeling and identification of the LpxC interactome lend support to the notion that FAB and FAD pathways modulate LpxC levels, as the overproduction of FabA, FabD and FabF leads to the destabilization of LpxC [65,89,93]. Thus, high amounts of flux towards saturated FAB increase LpxC proteolysis, and conversely, a *fabI* Ts mutant under non-permissive growth conditions leads to the increased flux in unsaturated FAB, which results in the enhanced stability of LpxC [14]. In addition to known genes involved FAB and FAD, it was also shown that the overproduction of the LPS-modifying enzyme WaaH, which transfers GlcUA to HepIII, also destabilizes LpxC [65]. The WaaH-dependent modification of HepIII occurs exclusively upon PhoB/R induction, when PhoB/R are constitutively expressed and upon entering the stationary phase [11]. Under stationary-phase conditions (slow-growth conditions), LpxC is known to rapidly degrade, which can link these pathways. Excess WaaH could also titrate a common metabolic precursor that is involved in GlcUA and L-Ara4N synthesis, which can trigger alterations in lipid A, and inner core modifications could give a signal to LpxC degradation to synthesize less LPS. Interactome studies also revealed that overexpression of *pyrH* gene-encoding UMP kinase destabilizes LpxC [65]. However, it is not known how PyrH levels modulate LpxC stability, although it is possible it could be due to the regulation of the UDP-GlcNAc precursor, which is used in the initial step of LPS biosynthesis. Finally, it needs to be emphasized that LpxC stability is highly dependent on levels of acyl-ACP since the biosynthesis of lipid A and phospholipids obtains its fatty acid chains from the (*R*)-3-hydroxyacyl-ACP pool [14,15]. Acyl-ACPs have also been implicated in the allosteric feedback regulation of fatty acid synthesis by inhibiting acetyl-CoA carboxylase and FabH. A diversion that can result in the competition of acyl-ACPs in different pathways, which can dramatically impact phospholipid and LPS biosynthesis and further lipid A biosynthesis, includes four different ACP-dependent acyltransferases (LpxA, LpxD, LpxL and LpxM) for the incorporation of fatty acids to generate hexa-acylated lipid A. Thus, the balanced usage of acyl-ACPs in glycerophospholipids and lipid A, which is regulated by FabZ and LpxC, is essential for bacterial viability, and in these pathways, the stability of LpxC plays a critical role. Consequently, the utilization of ACP as an intermediate in these pathways and if any additional modular protein exists needs to be identified.

## 13. MsbA Flippase Acts as a Major Checkpoint to Prevent Translocation of Underacylated LPS

**Figure 4:**
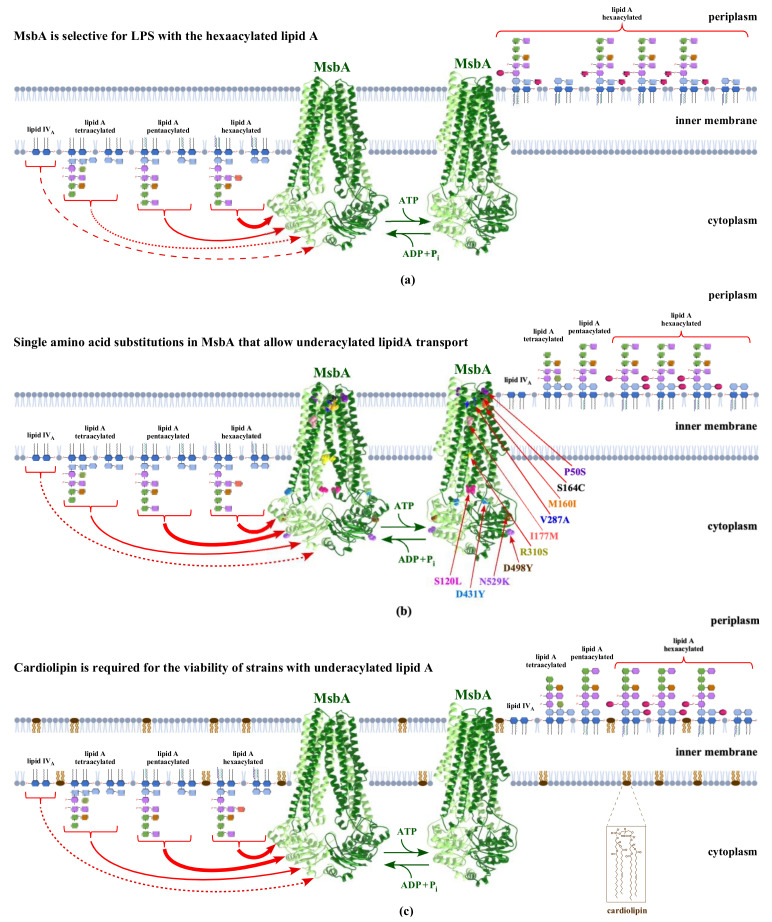
MsbA provides the crucial checkpoint by preferably selecting LPS with hexa-acylated lipid A. (**a**) MsbA has higher selectivity for hexa-acylated lipid A shown with a bold arrow. Selectivity for lipid IV_A_ or tetraacylated lipid A is highly reduced, depicted as dashed and thinner arrows. (**b**) Single amino acid substitutions mapping to the lipid A-binding domain or in the LPS exit groove of MsbA that allows the translocation of tetra-acylated Δ(*waaA*) or penta-acylated lipid A in Δ(*clsA lpxM*) translocation due to relaxation in hydrocarbon ruler properties. (**c**) Absence of cardiolipin is lethal when *E*. *coli* synthesizes underacylated LPS, which can be overcome by mutations in the *msbA* gene. Bold arrows indicate higher selectivity for hexa-acylated as compared to thinner or dashed arrows for tetra-acylated species. Inset shows the presence of cardiolipin.

A role for MsbA in LPS trafficking started emerging once it was shown that tetra-acylated lipid A species that is predominantly in *lpxL* mutants is poorly exported to the OM (Figure 4). Thus, strains either lacking LpxL or its derivatives lacking all three late acyl transferases Δ(*lpxL lpxM lpxP*) accumulate lipid IV_A_ precursors in the IM, and this defect can be overcome when MsbA is overexpressed (Figure 4a) [19,20,94]. The overexpression of *msbA* did not change the acylation pattern, but a significant fraction of tetra-acylated lipid A species that otherwise accumulate in *lpxL* mutants in the IM was transported to the OM [19]. Furthermore, *msbA*-deficient strains were shown to accumulate hexa-acylated lipid A species in the IM, further strengthening a role for MsbA in the transport of LPS [19]. In subsequent biochemical studies with MsbA-containing liposomes, the utilization of a Ts MsbA variant known as A270T with the impaired ATPase activity established that MsbA is a lipid-activated ATPase, and hexa-acylated lipid A is an especially potent activator [95]. Further support for MsbA flippase activity came from observations of the absence of lipid A modifications in a *msbA* missense mutant that occur only after translocation across the IM [96]. Subsequently, MsbA-dependent lipid A flippase was shown with the reconstitution of purified MsbA into proteoliposomes of *E*. *coli*, which required ATP hydrolysis [97]. 

Once it was established that MsbA acts as an LPS flippase translocating LPS across the IM to the periplasmic side, the suppressor analysis of strains synthesizing tetraacylated lipid species mapping to the *msbA* gene helped to establish that it serves as a key checkpoint in selectively reducing the transport of such precursor species. Thus, several suppressors of Ts phenotype of Δ(*waaC lpxL lpxM lpxP*) strains that synthesize LPS composed of only Kdo_2_- lipid IV_A_ or Δ(*lpxL lpxM lpxP*) strains with an intact core, but with lipid IV_A_, LPS was shown to map to either ATPase or lipid A-binding domains of MsbA [20]. Lipid A of such strains was found to be modified by phosphoethanolamine (*P-EtN*) but not by 4-amino-4-deoxy-L-arabinose (L-Ara4N), unlike the wild-type strains that showed both modifications. These results again reveal that tetraacylated lipid A is a poor substrate of MsbA, and the incorporation of L-Ara4N serves as a stringent marker for efficient LPS translocation of hexa-acylated LPS [20]. Moreover, the overexpression of *msbA* in Δ(*lpxL lpxM lpxP*) resulted in the incorporation of L-Ara4N when such strains were cultivated in a growth medium that induces lipid A modifications [20]. Even more compelling evidence was provided in the recent isolation and characterization of extragenic suppressors of either Δ*waaA* or Δ(*gmhD*-*waaA*) strains that mapped to the *msbA* gene [21]. Among the repeatedly isolated suppressors, two prominent single amino acid exchanges identified either P50S or R310S, allowing the efficient growth of such strains at elevated temperatures. The Pro50 amino acid residue is situated in the periplasmic groove predicted to be in contact with lipid A (Figure 4b). R310 amino acid is located in TM6, which is rich in positively charged and polar residues [21]. In the structure of MsbA in a complex with LPS, R310 could interact with phosphorylated glucosamine groups of lipid A and is part of the structure predicted to impart carbon chain ruler properties to MsbA (Figure 4) [21,98,99,100]. These amino acid alterations P50S and R310S in MsbA in Δ*waaA* derivatives were found to not only allow the growth in a rich medium at elevated temperatures but also allow the incorporation of modifications that occur only after LPS translocation, such as *P-EtN* and the addition of palmitate chains in the lipid A [21]. Thus, these suppressor mutations could alter carbon chain ruler properties and confer relaxed specificity to MsbA to translocate underacylated lipid A, more efficiently overcoming the barrier checkpoint exerted by the wild-type MsbA (Figure 4b).

Several structures of MsbA have been resolved with or without ATP, revealing a dimeric structure. In the initial studies, MsbA structures were obtained with proteins solubilized in detergent micelles without the presence of LPS [101]. This led to the model that MsbA in the absence of ATP is open facing the cytoplasmic side. However, in the presence of nucleotide, MsbA closes on the cytoplasmic side and opens on the periplasmic side of IM, leading to an inward-outward model [101]. Most revealing structures that elucidate the carbon chain ruler and how MsbA can be selective for C-12 and C-14 acyl chains came from studies that had LPS bound and also in the presence of specific inhibitors [98,99,100]. In one of the studies, cryo-electron microscopy imaging of MsbA in nanodiscs was employed, yielding a ‘trap and flip’ model of LPS translocation [98]. In this model, ADP-bound MsbA or in the absence of nucleotide exists in an open inward-facing conformation in the cytoplasm that opens TMDs to allow LPS to enter its chamber, aligning NBDs for ATP binding. This further leads to conformational changes in MsbA that facilitate the acyl chains to enter the periplasmic leaflet accompanied by MsbA rearrangement and ATP hydrolysis, thus leading to LPS translocation [98]. Other studies have used facial amphiphile FA-3 to obtain an active state with a structure displaying an inward-facing conformation similar to the cryo-EM structure [99,100]. Thus, lipid A-binding sites were found both inside the protein cavity and also on the outer surface cleft [100]. In this study, the inward-facing MsbA shows two portals on opposite sides made by TM4 and TM6 constituting the entry site of lipid A. These portals contain a stretch of positively charged and polar residues that could interact with negatively charged residues in LPS [100]. These residues include some of the amino acids described above, such as R310, which was identified to relax the specificity of MsbA toward recognition of tetraacylated lipid A precursor [21]. In the same structural study, authors identified a putative lipid A-binding site situated above the shallow surface groove formed at the periplasmic ends of TM1, TM2 and TM3, which could constitute the exit portal for lipid A [100]. In support of these structural studies of the location of lipid A at the exit site, amino acid exchanges, such as P50S, M160I and S164C in MsbA at this groove, also allow the translocation of tetra- and penta-acylated lipid A species, as judged by the suppression of growth defects of various strains synthesizing underacylated lipid A (Figure 4b) [21]. The explanation for MsbA selectivity for hexa-acylated lipid A and selecting against underacylated and longer acyl chains has come from additional structural studies wherein the structure of MsbA in a complex with small molecule inhibitors that either occupy the substrate-binding site or alter NBD distance and affect ATP hydrolysis [99,102]. One of these inhibitors (TBT1) binds in the substrate-binding site, leading to a distortion in the conformation of MsbA, which causes a decreased distance in NBDs while hijacking the LPS-binding position [102]. On the other hand, the binding of two molecules of another inhibitor G247 increases the NBD distance in a wider inward state [102]. TBT1 binds the same charged residues that LPS binds in TM2 and TM6; however, its binding moves TM6 into the center of the inner cavity, leading to the collapse of TMD2 around TM6. Analysis of the G507 compound, which is related to G247, in a complex with MsbA provided further insights into why MsbA confers the selectivity toward hexa-acylated lipid A rather than tetra-acylated species [99]. In the structure of the G092–LPS–MsbA ternary complex, it was found that the inner vestibule of MsbA forms a thimble-like structure, functioning as a hydrocarbon ruler using its charged Arg residues to select for C-12 and C-14 acyl chains with reduced selection for longer acyl chains [99].

## 14. Cardiolipins Are Required for the Viability of Strains Synthesizing Underacylated LPS, Thus Providing Another Checkpoint

As mentioned in the above sections, the OM of Gram-negative bacteria is asymmetric, and the maintenance of this asymmetry is crucial for bacterial viability. The inner leaflet of OM contains glycerophospholipids. In bacteria such as *E. coli*, phospholipids are made up of phosphatidylethanolamine (PE), phosphatidylglycerol (PG) and cardiolipin (CL) in an approximate ratio of 75:20:5 [103]. A functional role for CL in relation to LPS had not been established until recently [21]. *E. coli* contains three genes: *clsA*, *clsB* and *clsC*, whose products are involved in the CL synthesis. Among these genes, transcription of the *clsA* gene is induced under a variety of stress conditions, and its product ClsA is the major cardiolipin synthase [104,105,106]. Recent studies showed that ClsA plays an essential role in strains synthesizing underacylated lipid A and is also required for the DksA-mediated multicopy suppression of strains that lack the six major cytoplasmic peptidyl-prolyl *cis*/*trans* isomerases (Figure 4c) [21,22,107]. A role for CL in Δ*waaA* strains synthesizing glycosylation free lipid IV_A_ was hinted at because CL was found to prominently accumulate in such backgrounds, as shown by the mass spectrometric analysis of a mixture of lipid A and glycerophospholipids [20,21]. Furthermore, CL species were also prominent in the spectra of Δ(*lpxMP*). To further investigate if ClsA is required for the growth of strains that synthesize underacylated LPS, genetic studies revealed that a Δ*clsA* mutation could not be introduced in either Δ*waaA* or Δ(*lpxLMP*) backgrounds even under permissive growth conditions. To investigate which of the three acyltransferases is required, it was shown that Δ(*clsA lpxL*) is also lethal under conditions when LpxL is not required for bacterial viability, while the Δ(*clsA lpxM*) combination is conditionally lethal. Thus, a Δ(*clsA lpxM*) derivative turned out to be lethal at 37 °C or above but is viable at 30 °C. Based on these genetic results, it was concluded that underacylated lipid A species, which are poorly translocated by MsbA, require ClsA for LPS translocation to the OM, thus constituting another checkpoint in the LPS synthesis (Figure 4c). This was supported by the saturated isolation of extragenic suppressors of Δ(*clsA lpxM*). The majority of such suppressors were mapped to MsbA, with most of them mapping to either the NBD domain, in the groove predicted to be involved in LPS exit or in the portals constituting TM4 and TM6 (Figure 4b). All amino acid exchanges in MsbA that suppress Δ(*clsA lpxM*) map to positions shown to be contact regions with LPS or with drug molecules that highjack substrate binding of MsbA based on recently available structures [98,99,100,102]. Another independent study also showed a critical requirement for CL in the Δ*lpxM* background, and depletion experiments revealed that a substantial amount of LPS is retained in the IM, causing the lethality [22]. Taken together, these studies suggest coordination between CL and MsbA in lipid A trafficking, which is critical when the lipid A is underacylated and also highlights the integration of phospholipid and LPS biosynthetic pathways in balancing the OM composition.

## 15. Checkpoints That Regulate Relative Abundance of LPS Glycoforms and Transcription of the Main *waaQ* Operon

The LPS composition is highly heterogeneous due to the non-stoichiometric incorporation of modifications in the lipid A part or in the inner core, which are subjected to regulation by the RpoE sigma factor and various TCSs, such as BasS/R, PhoP/Q and PhoB/R, as well as certain embedded sRNAs. In *E. coli* K-12, the commonly observed modification of the lipid A part arises due to the incorporation of *P-EtN* and/or L-Ara4N or changes in acylation and phosphorylation at the 1′ position in the reducing glucosamine unit, yielding *tri*-phosphorylated lipid A species (reviewed in [3,5]). Modifications by *P-EtN* and L-Ara4N are observed when BasS/R TCS is induced, which confers resistance to polymyxin B by masking negative charges in lipid A. The inner core can also be modified by the addition of a third Kdo moiety by WaaZ Kdo transferase, the incorporation of rhamnose (Rha) on either the second Kdo or the third terminal Kdo depending on the non-stoichiometric incorporation of an EptB-dependent modification of the second Kdo [9]. The inner core can also vary depending upon variations in the phosphorylation of heptose (HepI and HepII) residues, the incorporation of uronic acid, such as GlcN, and the truncation of the outer core terminal disaccharide, when either WaaR is limiting or when a third Kdo is incorporated [9]. Depending on which modifications or combinations occur, the relative abundance of various glycoforms is determined. In *E. coli* K-12, up to now, seven different glycoforms have been identified (see [3,5]), and here, we discuss only a few checkpoints that determine the switch to the synthesis of a particular glycoform. In rich medium under optimal growth conditions, glycoform I comprises about 70% of LPS with typical two Kdo residues linked to lipid A and the normal inner and outer cores [108]. However, three other minor glycoforms were also observed, including glycoform IV, which contains a branched tetrasaccharide linked to lipid A, with a third Kdo and Rha [108]. When *E. coli* K-12 is grown in growth medium that is BasS/R- and PhoB/R-inducing, a profound shift to the accumulation of glycoforms with a third Kdo and Rha are observed at the expense of glycoform I, resulting in glycoform IV, V and VII synthesis. Glycoforms IV and V are similar except for the incorporation of *P-EtN* on the second Kdo and Rha attached to the terminal third Kdo instead of the second Kdo in glycoform V. Glycoform VII has additional GlcUA attached to HepIII with a concomitant absence of phosphate on the HepII [9,11]. All these glycoforms with the third Kdo have a truncation of the outer core terminal disaccharide [9,108]. The synthesis of glycoforms with the third Kdo with *P-EtN* incorporation is also a signal of inducing the envelope stress response under the control of the RpoE sigma factor. Several factors determine these switches: (i) strains that synthesize tetraacylated lipid A due to the absence of three late acyltransferases with intact cores primarily accumulate glycoforms with a third Kdo under lipid A-modifying growth conditions [20]. However, the overexpression of the *msbA* gene restores the synthesis of glycoforms with two Kdo residues, suggesting a preference for the synthesis of glycoforms with the third Kdo when LPS is poorly translocated. (ii) Under RpoE-inducing conditions, such as in Δ*rseA* strains, glycoform V and its derivatives with a third Kdo and *P-EtN* on the second Kdo are synthesized near exclusively due to the transcriptional induction of the *eptB* gene and overcoming translational repression of *eptB* mRNA by the MgrR sRNA. (iii) The truncation of the outer core under RpoE-inducing conditions is ascribed due to translational repression of *waaR* mRNA by the RybB sRNA; thus, WaaR levels determine the checkpoint in the incorporation of glycoforms with a third Kdo. (iv) The molecular switch to the synthesis of glycoforms with a third Kdo also requires ppGpp and transcriptional upregulation of the *waaZ* mRNA [9]. (v) Analysis of the LPS structure of strains that individually lack LPS biosynthetic genes revealed that the main branch point that determines these glycoform switches is the synthesis of Kdo_2_Hep_2_Hex_2_ (with HepI phosphorylated), where the second hexose can be either Gal or Glc since Δ*waaO* mutants can incorporate Kdo_3_Rha, but Δ(*waaB-waaO*) strains cannot [9]. 

Regarding the prevention of excess LPS synthesis and the regulation of large *waaQ* operon’s expression, which contains several genes encoding various LPS biosynthetic enzymes, another checkpoint is exerted by the RirA sRNA. RirA is a 73-nt sRNA located in the 5′UTR of the *waaQ* operon [23]. Transcription of the *waaQ* and *rfb* operons requires binding RfaH to an 8-nt sequence GGCGGTAG in the 5′UTR called the *ops* (operon polarity suppressor) pause site [24,109,110]. RfaH is a paralog of a universally conserved family of transcription factors represented in *E. coli* by NusG, which is a bona fide transcription elongation factor [111]. The presence of the *ops* pause site and its recognition by RfaH distinguishes it from NusG and confers the specificity for the transcription of the *waaQ* operon, and this recruitment prevents transcriptional termination and enhances transcriptional elongation, ensuring LPS biosynthesis [112]. RfaH is a fold-switching protein, and in the absence of interaction with the *ops* site, it exists in an autoinhibited conformation, wherein its RNAP-binding NTD site is masked by its helical CTD. However, upon encountering *ops*-paused RNAP, RfaH undergoes a conformation switch with its CTD refolding into β-barrel that recruits S10, thereby coupling transcription with translation and enhancing transcription elongation [112]. RirA sRNA binding to RfaH in the presence of RNAP could, at this point, counterbalance RfaH-mediated transcription of *waaQ* and *rfb* operons either by titrating RfaH or by rendering RfaH into its autoinhibited state. It is worth mentioning that RirA interacts with RfaH in the presence of RNAP, and the overexpression of RirA sRNA mimics a Δ*rfaH* phenotype (abrogation of *O*-antigen incorporation, reduction in LPS amounts and truncation of LPS) [23]. This action by RirA could serve as a checkpoint to prevent excess biosynthesis of LPS since *rirA* and *waaQ* mRNA share the same transcription start site [23]. 

## 16. Critical Role of RpoH, RpoE and Two-Component Systems in Regulating LPS Biosynthesis

**Figure 5:**
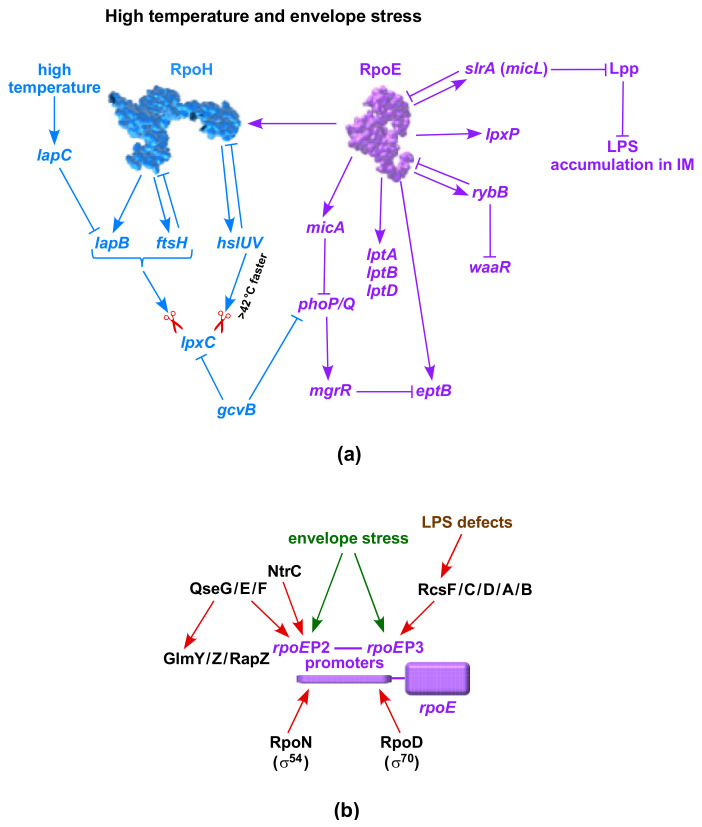
Alternative sigma factors RpoH and RpoE induced at high temperatures and envelope stress, respectively, control several essential processes in LPS biosynthesis. (**a**) The expression of LapC, LapB, FtsH and HslVU regulating LpxC turnover are induced at high temperatures. Genes encoding LapB, FtsH and HslVU are members of the RpoH regulon. RpoE is required for the transcription of several genes involved in LPS biosynthesis and also activates the transcription of the *rpoH* gene at high temperatures. sRNAs MicA, RybB and SlrA constitute the non-coding arm of the RpoE regulon. MicA and RybB are involved in the regulation of LPS modifications. The overproduction of SlrA represses the Lpp synthesis, thereby relieving the accumulation of toxic buildup of LPS in the IM in the absence of LapB and thus repressing the hyperinduction of RpoE in such backgrounds. (**b**) Severe envelope stress due to LPS defects induces the transcription of the *rpoE* gene via the induction of *rpoE*P2 and P3 promoters, particularly *rpoE*P3, which requires Rcs system activation.

Crucial roles exerted by LapB, FtsH and LapC in the regulation of LpxC are now established beyond a reasonable doubt. Importantly, the transcription of genes encoding these three essential proteins is induced at high temperatures [15,38]. At the transcriptional level, one of three promoters of the *lapAB* operon and that of the *ftsH* gene are recognized by RNAP complexed with the RpoH heat shock sigma factor (Figure 5). Analysis of a Δ*rpoH* strain’s LPS showed the accumulation of prematurely synthesized LPS species, although this defect is not as severe as observed for a Δ*lapB* derivative [15]. RpoH-regulated major heat shock proteins DnaK and DnaJ also co-purify with LapA and LapB proteins, and Δ(*lapAB dnaKJ*) exhibits more severe LPS defects, including the accumulation of penta-acylated lipid A species [15]. Thus, RpoH exerts major control over regulating LpxC turnover, and heat shock proteins (DnaK and DnaJ) could play a role in the delivery of some of LPS biosynthetic enzymes to LapB for proper LPS assembly. This is further supported by the suppression of some of the phenotypic defects by the overexpression of *dnaK/J* genes [15]. Regarding induced transcription of the *lapC* gene at high temperatures, the molecular basis is not clear as it is not a member of the *rpoH* regulon [38]. Of significance, the transcription of RpoH-regulated genes is also induced in a Δ*lapA/B* strain. This induction of *rpoH* could be a consequence of the accumulation of misfolded LPS biosynthetic enzymes, such as LpxM, WaaC and WaaO, in the absence of LapB [15]. To ensure the increased LPS synthesis at high temperatures, the transcription of the *gmhD* (*htrM*) operon cotranscribed with *waaC* and *waaF* genes is induced at elevated temperatures [113]. This transcriptional upregulation would allow enough molecules of early heptosyltransferases to be available as per the demand of LPS synthesis. Since LPS demand is higher under fast-growing conditions, protein-folding machinery has to be optimally tuned for the availability of correctly folded LPS biosynthetic enzymes. A requirement of protein-folding catalysts for the folding of many such enzymes has been recently reported, as in the absence of such catalysts, some lipid A biosynthetic enzymes were found to aggregate [114].

Based on the hyperinduction of the *rpoE* gene’s transcription, the genetic basis for isolating mutations in *lapAB* and *lapC* genes is in line with an established dogma that envelope stress caused by LPS defects is sensed and regulated by this sigma factor [3,115]. The transcription of the *rpoE* gene is initiated from six promoters, with the most proximal P6 promoter recognized by Eσ^E^ itself and P2 and P3 promoters sharing the same start site [23]. Although transcription initiated from P2, P3 and P6 promoters is induced upon defects in LPS, most striking is the pronounced induction of the P3 promoter, which requires the Rcs two-component system as the activator (Figure 5) [23]. Thus, mutations in *lapB*, *lapC*, *waaC* and *waaF* strongly induce transcription from the *rpoE*P3 promoter [23,38]. RpoE belongs to the ECF sigma factor family [116,117]. Thus, its regulon members include genes, whose products are involved in either protein folding or assembly of OM, including LPS transport and certain steps in the LPS biosynthesis and its modifications [7,115,118,119]. RpoE also transcribes genes encoding regulatory RNAs, such as MicA and RybB, which play important roles in repressing the OMP synthesis, and SlrA (MicL), which represses the Lpp synthesis (Figure 5) [82]. RybB and MicA are also involved in the regulation of LPS glycoform switches. MicA further represses PhoP, thereby linking RpoE with LPS modifications [120]. The *eptB* gene is also a member of the r*poE* regulon, whose product is required for the modification of the second Kdo by *P-EtN* and conferring polymyxin B resistance. Cpx TCS also overlaps with RpoE function, as they positively regulate the expression of *fabZ*, *lpxA* and *lpxD* genes [115]. In this circulatory regulation, RpoE positively regulates the transcription of *rpoH*P3, which is the main promoter that is active at high temperatures [116,121,122]. Consistent with a prominent role for the RpoE-mediated control of LPS biosynthesis, the transcription of *waaWVL* operon in the adherent-invasive *E*. *coli* strain associated with Crohn’s disease is regulated by Eσ^E^ polymerase [123]. Thus, RpoH and RpoE using some of their regulon members, along with TCSs, play essential roles in maintaining the OM integrity by regulating LPS biosynthesis at critical checkpoints.

## 17. Conclusions and Perspectives

The last year has seen tremendous progress in our understanding of the regulation of the first committed step in LPS biosynthesis through the discovery of LPS sensing in the periplasm by the essential LapC protein and how LapC inhibits LapB, thereby setting LapC degradation by FtsH as per the demand of LPS synthesis. Another key discovery is the finding that the HslVU (ClpQY) protease complex can degrade LpxC, particularly at high temperatures, independent of LapB–FtsH. The LpxC-mediated critical checkpoint can further be subjected to post-transcriptional control by the GcvB sRNA. MsbA was already known to exert another major checkpoint by exerting its hydrocarbon ruler function to preferentially select hexa-acylated lipid A species. Previously, a critical function for the cardiolipin synthesis in regulating the homeostasis of various OM components in *E. coli* was unknown. However, for the viability of strains synthesizing underacylated LPS that is poorly translocated, a new additional role for cardiolipin synthase (ClsA) was revealed very recently since Δ(*clsA lpxL*) and Δ(*waaA*-*clsA*) combinations are synthetically lethal and Δ(*clsA lpxM*) bacteria synthesizing penta-acylated LPS are conditionally lethal. The majority of suppressors of such conditional lethality of Δ(*clsA lpxM*) bacteria map to the *msbA* gene, identifying the critical residues involved in lipid A binding and LPS exit from MsbA. How MsbA and cardiolipins cooperate in LPS translocation is, at present, not understood. Thus, further studies are required to address this issue.

Most importantly, it is still unknown how LapC senses LPS and what is the precise regulatory mechanism of interaction between LapC and LapB that determines when the inhibitory action of LapC on LapB should be relieved. It is particularly pertinent since, in the absence of the periplasmic domain of LapC, LpxC is destabilized, while LapB and LapC TMD interactions still persist. Furthermore, the mechanism of GcvB-mediated control of LpxC needs to be established, and GcvB could act in an indirect manner in this process. Furthermore, it is not known which amino acid residues in LpxC or signals direct its degradation by HslVU, so these aspects need future studies. Similarly, additional elements or pathways, particularly alterations in fatty acid composition, and their role in controlling LpxC stability, which maintains a delicate balance between phospholipids and LPS, and how lipid A disaccharide is sensed require further molecular characterization.

It is important to mention here that certain bacteria, such as *Pseudomonas aeruginosa*, do not recruit FtsH to control LpxC levels, and it will be interesting to know which modes of LPS synthesis regulation exist in such important pathogenic bacteria [124]. Furthermore, in *Agrobacterium tumefaciens* and *Rhodobacter capsulatus*, LpxC proteolysis is mediated by Lon protease instead of LpxC [124].

As LPS constitutes a key virulence factor in pathogenic bacteria and many proteins involved in its biosynthesis and transport are unique in Gram-negative bacteria, they are validated targets for identifying new antibiotics. Thus, inhibitors of LpxC, such as CHIR090 and its derivatives, are known as potent antimicrobial compounds [63]. As mentioned in the section covering MsbA’s structural analysis, many inhibitors, such as G247, G092, G507 and TBT1, are potential new antibiotics as they inhibit LPS transport. Similarly, inhibitors of the Lpt transport system based on macrocyclic peptidomimetics and derivatives, such as Murepavadin, have been shown to be effective against *P*. *aeruginosa* [125]. These new strategies reveal their vast potential to combat emerging multidrug-resistant Gram-negative bacteria based on understanding the essential steps of LPS biosynthesis and its control.

## Figures and Tables

**Table 1:** Genes whose products are involved in Kdo_2_-lipid A biosynthesis.

Gene	EC Number	Product
*lpxA*	2.3.1.129	acyl-[acyl-carrier-protein]-UDP-*N*-acetyloglucosamine *O*-acyltransferase
*lpxB*	2.4.1.182	lipid A disaccharide synthase
*lpxC*	3.5.1.108	UDP-3-*O*-acyl *N*-acetylglucosamine deacetylase
*lpxD*	2.3.1.191	UDP-3-*O*-(3-hydroxyacyl)-glucosamine *N*-acyltransferase
*lpxH*	3.6.1.54	UDP-2,3-diacylglucosamine diphosphatase
*lpxK*	2.7.1.130	tetraacyldisaccharide A 4′-kinase
*lpxL*	2.3.1.241	Kdo_2_-lipid IV_A_ acyltransferase
*lpxM*	2.3.1.243	acyl-Kdo_2_-lipid IV_A_ acyltransferase
*lpxP*	2.3.1.242	Kdo_2_-lipid IV_A_ palmitoleoyltransferase
*lpxT*	2.7.4.29	Kdo_2_-lipid IV_A_ phosphotransferase
*waaA*	2.4.99.12/ 2.4.99.13	lipid IV_A_ 3-deoxy-α-D-*manno*-octulosonic acid transferase/ (Kdo)-lipid IV_A_ 3-deoxy-α-D-*manno*-octulosonic acid transferase

## Data Availability

Data are contained within the article.
